# Insulin depot absorption modeling and pharmacokinetic simulation with insulin glargine 300 U/mL 

**DOI:** 10.5414/CP203269

**Published:** 2018-10-26

**Authors:** Klaus Lindauer, Reinhard Becker

**Affiliations:** Sanofi-Aventis Deutschland GmbH, Industriepark Hoechst, Frankfurt am Main, Germany

**Keywords:** Gla-300, Gla-100, insulin depot absorption, mechanistic pharmacokinetic simulation, modeling

## Abstract

Objective: Mathematical models of insulin absorption have been used to predict plasma insulin concentrations after administration, but few are specifically applicable to insulin glargine, which precipitates subcutaneously after injection. Materials and methods: The formation and redissolution of subcutaneous depots of insulin glargine 100 U/mL (Gla-100) and insulin glargine 300 U/mL (Gla-300) are modeled. Surface-area-dependent redissolution is introduced to established diffusion and absorption pathways, and pharmacokinetic (PK) profiles are simulated and subsequently validated using experimental data from euglycemic glucose clamp studies. Simulations are used to predict the PK effect of adapting the timing of once-daily insulin injections and of switching from one insulin product to the other. Results: Simulated PK profiles resemble those previously observed in clinical trials, with Gla-300 providing more gradual and prolonged release of Gla-300 vs. Gla-100, owing to a more compact depot. The predicted PK profile of Gla-300 shows less fluctuation in plasma insulin concentrations than that of Gla-100, and may be better suited to adapting the timing of daily injections to account for variation in daily activities. Simulating a switch from one insulin glargine product to the other results in temporary alteration of previous steady state, but this is regained within ~ 3 days. Conclusion: This study suggests that PK differences between Gla-300 and Gla-100 are a product of the more compact Gla-300 depot and its smaller surface area. The model employed also allowed estimation of insulin glargine concentrations when varying the time interval between injections as well as when switching from one insulin glargine product to the other.

## Introduction 

Mathematical models pertinent to the absorption of subcutaneously injected insulin products have been developed to predict plasma insulin concentrations [1, 2]. Such models have been used to surmise absorption of aggregates of insulin molecules remaining soluble after injection and of preformed crystals [3], as well as mixtures of the two [4]. However, only a few studies directly address absorption from a subcutaneous (SC) precipitate, as applicable to insulin glargine [5, 6]. 

Insulin glargine 300 U/mL (Toujeo (Sanofi, Paris, France); Gla-300) contains the same active molecule as insulin glargine 100 U/mL (Lantus (Sanofi); Gla-100) [7], delivering the same number of insulin units but in 1/3 of the injection volume. Euglycemic glucose clamp studies in people with type 1 diabetes (T1DM) have demonstrated that Gla-300 provides more even and prolonged pharmacokinetic (PK) and hence pharmacodynamic (PD) profiles than Gla-100 [8], and low levels of within- and between-day PK fluctuation [9]. In randomized clinical trials in which basal insulin dose was titrated seeking a predefined self-monitored plasma glucose target, this translated into less hypoglycemia [10, 11, 12, 13, 14, 15, 16] and apparently lower glycemic fluctuation with the same efficacy in terms of HbA_1c_ reduction [17]. 

Both Gla-300 and Gla-100 are injected as unbuffered acidic solutions (pH 4). However, as insulin glargine is isoelectric at physiological pH, it precipitates after SC injection, forming an amorphous depot [18, 19]. Gradual redissolution from this SC depot is the key retardation principle. 

The difference in PK and PD profiles between these two long-acting insulin glargine-based products is thought to rest in the different SC depot size, resulting in different rates, and therefore durations, of release for a given administered dose. Therefore, the more compact depot of Gla-300, compared with that of Gla-100, leads to more gradual and prolonged redissolution, as observed in previous studies [8, 20]. 

Redissolution occurs at the boundary area between the SC depot and the adjacent tissue. Therefore, according to the Nernst-Brunner equation [21], the surface area of the depot and insulin glargine solubility at physiological pH should be the primary determinants of insulin glargine release. Given the relationship between the volume and surface area of a geometric body, as a Gla-300 depot will be ~ 1/3 of the volume of a Gla-100 depot, it will have approximately half the surface area, which should result in a substantially reduced redissolution rate [22]. This relationship holds for any form of depot, although the surface area to volume ratio will vary for different depot shapes and therefore influence the absolute redissolution rate. The actual shape of an insulin glargine depot is unknown, but it has been shown that soluble insulin depots deviate from being spherical because the injected insulin will follow the septal space and pressure between adipose cells [23, 24] and this may also apply to insulin glargine. 

The above considerations concerning insulin glargine depot formation and redissolution were introduced to modify existing mathematical models in order to simulate the differences in PK profiles of Gla-300 and Gla-100 observed in the euglycemic clamp studies. The model adds surface-area-dependent redissolution of hexameric insulin glargine from amorphous single-bodied precipitates to defined diffusion kinetics and absorption pathways of di- and monomeric insulin glargine entities. 

In addition, we illustrate potential applications for such simulated profiles by predicting the PK effects of altering the time interval between insulin glargine administrations in a once-daily regimen and of switching from one insulin glargine product to the other. 

Basal insulin substitution affects control and disposition of hepatic glucose production, which requires plasma insulin concentration of ~ 10 µU/mL (or 60 pmol/L) in patients with T1DM [25, 26]. This is achieved with approximately, or slightly less than, 0.4 U/kg (or 2.4 nmol/kg) of insulin glargine (either Gla-100 or Gla-300). Higher doses, such as 0.6 U/kg and above, may serve to characterize dose exposure-response relationships for glucose utilization, but produce strong hyperinsulinemia, requiring massive compensatory glucose loads [27]. In contrast, actual therapeutic doses used to control fasting, and hence hepatic glucose production, in particular in type 2 diabetes, are subject to an individual’s body weight, insulin sensitivity, bioavailability, and concomitant medication. 

The focus of simulations therefore rests on describing PK of physiologically relevant doses and concentrations modeled for an average subject. 

## Materials and methods 

### Observed pharmacokinetics 

The modeling and simulation in this article is based on PK of euglycemic clamp studies in people with T1DM. The first of these studies (study 1) was a double-blind, randomized crossover study involving 24 people of European ancestry aged 18 – 65 years, who received single SC doses of Gla-300 (0.4, 0.6, and 0.9 U/kg (2.4, 3.6, and 5.4 nmol/kg)) and Gla-100 (0.4 U/kg (2.4 nmol/kg)) followed by a 36-hour clamp procedure [20]. The second study (study 2) tested single SC doses of Gla-100 (0.4 U/kg (2.4 nmol/kg)) and included 26 people with T1DM undergoing a 24-hour clamp procedure (data on file). 

### Modeling and simulation details 

The model below assumes that by far the majority of insulin glargine precipitates as a coherent amorphous single-bodied SC depot before being redissolved and released as hexamers, subsequently dissociating into dimers and monomers, spreading into the SC tissue and being absorbed into the blood. In addition, a small amount of soluble insulin glargine is assumed to be immediately absorbed after SC injection. While residing in SC tissue, glargine, like any other insulin, is subject to non-specific loss by degradation [4], which affects bioavailability. The rapid conversion of monomers to fully soluble 21^A^-Gly-human insulin (metabolite M1, the primary molecule responsible for the metabolic effect of insulin glargine) is not captured in this model. However, this should not affect the validity of the model as insulin glargine metabolism is the same with both Gla-300 and Gla-100, with almost all insulin glargine monomers being converted into metabolite M1 [7]. 

**Formation and redissolution of the SC depot**

The formation of the depot is described by Equation 1, where *pw* denotes the fraction of solution vs. precipitate at the surface of the depot and *k* *_FP_* is a constant representing the rate of precipitate formation. 


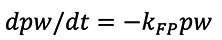
 (Equation 1) 

The redissolution rate of a particular Gla-300 or Gla-100 depot at time *t *(*dGla* *_Depot_*/*dt*), where *Gla* *_Depot_* represents the amount of insulin glargine within the depot, is a diffusion-driven process and is dependent upon both the surface area of the precipitate and a constant describing the rate of redissolution of an insulin glargine precipitate (*k* *_pre_*). Details of the full parameter set used for modeling and simulation are given in Table 1. Assuming a single-bodied depot, its surface area (*SA*) can be expressed in terms of the depot volume corrected by *f* *_vol_* for sphericity, the deviation of the depot shape from a sphere as the injected volume spreads within the SC tissue 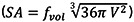 , as the precipitate shape is defined by the elasticity of the SC tissue [23]. Since the concentration of an insulin glargine formulation (*C* *_Formulation_*) defines its volume for a given dose, the depot volume at time *t* can be expressed as (*Gla* *_Depot_*/*C* *_Formulation_*). Therefore, the overall redissolution rate of an insulin glargine depot can be expressed as shown in Equation 2, which also accounts for the small amount of insulin glargine that will be absorbed at a similar rate to that of soluble insulin (rate *k* *_sol_*) and the change in depot mass over time. 


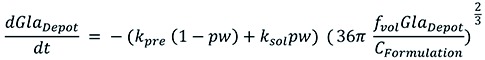
 (Equation 2) 

**Diffusion of insulin glargine within SC tissue and dissociation of oligomers**

Once redissolved from the precipitate body, the dissociation of insulin glargine hexamers (*H*) into dimers (*D*) and monomers (*M*), and the subsequent spatial spread (V_sc_) into the SC tissue is also captured by a diffusion-controlled process, as described in Equations 3 – 5: 


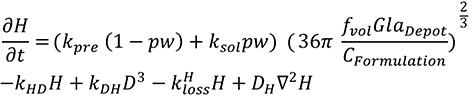
 (Equation 3) 


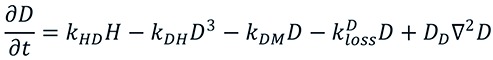
 (Equation 4) 


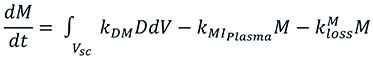
 (Equation 5) 

*D* *_H_* and *D* *_D_* symbolize the diffusion constants of *H* and *D,* respectively, whereas 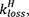 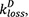 and 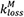 represent the non-specific loss of the *H*, *D,* and *M* species in the SC tissue. The kinetic constants *k* *_HD_*, *k* *_DH_*, and *k* *_DM_* represent the dissociation process of *H* to *D* and *M*. The transition of insulin glargine into the blood is captured by 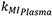 and *V* *_SC_* is the SC volume in which insulin glargine is spread. 

**Distribution of insulin glargine in the blood and clearance**

The molar amount of insulin glargine in the blood (*Gla* *_Plasma_*) depends on the amount absorbed from the SC tissue, the estimated plasma distribution volume, *V* *_Plasma_* and the amount cleared from the blood. *V* *_Plasma_* is dependent on body mass (BM) (*V* *_Plasma_* = *V* *_D_* *× BM,* where *V* *_D_* **= 0.1421 L/kg), therefore the amount of insulin glargine in the blood at a given time is described by Equation 6. 


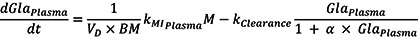
 (Equation 6) 

**Parameter estimation and validation**

In order to estimate the values of the parameters influencing the formation and redissolution of the SC depot (*k* *_FP_*, *k* *_pre_*, and *k* *_sol_*), the transition of insulin glargine (*k* *_HD_* and *k* *_DM_*) and the non-specific loss within the SC tissue (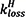), Gla-100 data from a previous single-dose study (study 1) [20] were used to evaluate and validate the prediction accuracy. The fitting routine for these parameters was repeated multiple times by varying the initial parameter settings. Consequently, multiple solutions were obtained, and altered by the mean squared error (MSE) of the plasma insulin glargine concentration value calculated by using the respective values at the following time points: 6, 8, 12, 16, 20, 24, and 28 hours after administration. The resulting mean parameter set is listed in Supplemental Table 1. For the final parameter set, the resulting expected plasma profile was calculated and compared with the average Gla-100 profile from study 1 [20]. 

The model was validated using the average Gla-100 concentration profile from a separate independent single-dose study (study 2). Additionally, the model was compared against the average Gla-300 concentration profiles for various doses (0.4, 0.6, and 0.9 U/kg (2.4, 3.6, and 5.4 nmol/kg)) from study 1. The calculated MSE values are listed in Supplemental Table 2. 

### Simulation application 1: Effect of a flexible dosing regimen 

As one example of the application of the model already described, we used the simulated PK profiles of Gla-300 and Gla-100 to predict the effect of varying the interval between once-daily injections. A steady-state scenario, based upon a person with T1DM (80 kg body weight) receiving Gla-300 or Gla-100 once daily, was initially generated by assuming that injections of insulin glargine were administered every 24 hours until steady state was achieved. The time between injections was then varied using a number of different scenarios, as shown in Supplemental Table 3. The resultant effect on both PK profiles and 24-hour insulin glargine exposure (area under the insulin concentration-time curve in the 24 hours prior to a given time point *t* (AUC*_t_*_–24 to_ *_t_*)) is displayed graphically. Gla-300 and Gla-100 have slightly different bioavailability, which is thought to be an effect of the more gradual release of Gla-300 from the SC depot, resulting in greater local degradation. In line with this, 24-hour insulin exposure is lower with Gla-300 than with Gla-100, for the same SC injected molar amount [8]. In order to better visualize the relative effect of the flexible dosing regimen, the dose of Gla-300 was therefore set to 0.432 U/kg (2.593 nmol/kg) vs. 0.4 U/kg (2.4 nmol/kg) with Gla-100, an 8% increase with Gla-300 that harmonizes the 24-hour exposure with both insulin products in this model. 

### Simulation application 2: Switching from one insulin glargine product to the other 

As a second application of the modeling and simulation, we predicted the PK effect of switching from one insulin glargine product to the other (Gla-300 to Gla-100 and vice versa) once steady state is achieved by a once-daily regimen with 24-hour injection intervals. The dose of each treatment was set as previously stated, to visualize the relative effect of switching. This also reflects recommendations for clinical practice whereby the daily insulin dose may have to be altered when switching from one insulin to the other [28, 29]. Results are presented graphically, as for simulation application 1. 

### Software 

Computations were performed in Matlab (version R2013b (8.2.0.701), MathWorks, MA). For parameter estimation the OPTI Toolbox (Version 2.05) was applied, which solves the nonlinear least squares problem by utilizing the NOMAD Blackbox Optimization Software (Version v3.6.2) [30, 31]. 

## Results 

### Modeling and simulation 

**Figure 1.**
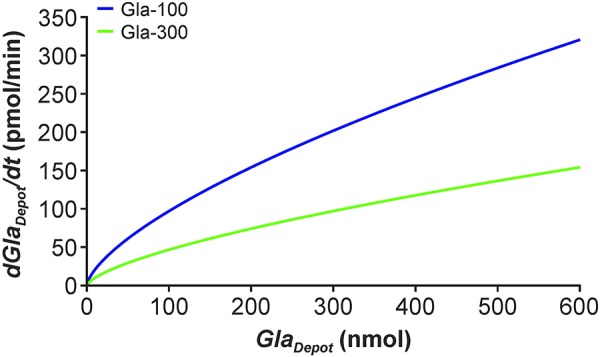
Estimated redissolution rate (*dGla* *_Depot_*/d*t*) of Gla-300 and Gla-100 depots with changing depot size (represented by *Gla* *_Depot_*, the amount of insulin glargine within the depot).

Figure 1 shows the lower redissolution rate of Gla-300 compared with Gla-100 as a result of the reduced surface area of the Gla-300 depot. As anticipated, the redissolution rate of Gla-300 is approximately half that of Gla-100, in line with the reduced depot surface area. This plot also demonstrates how redissolution rate alters with changing depot mass and size. 

**Figure 2.**
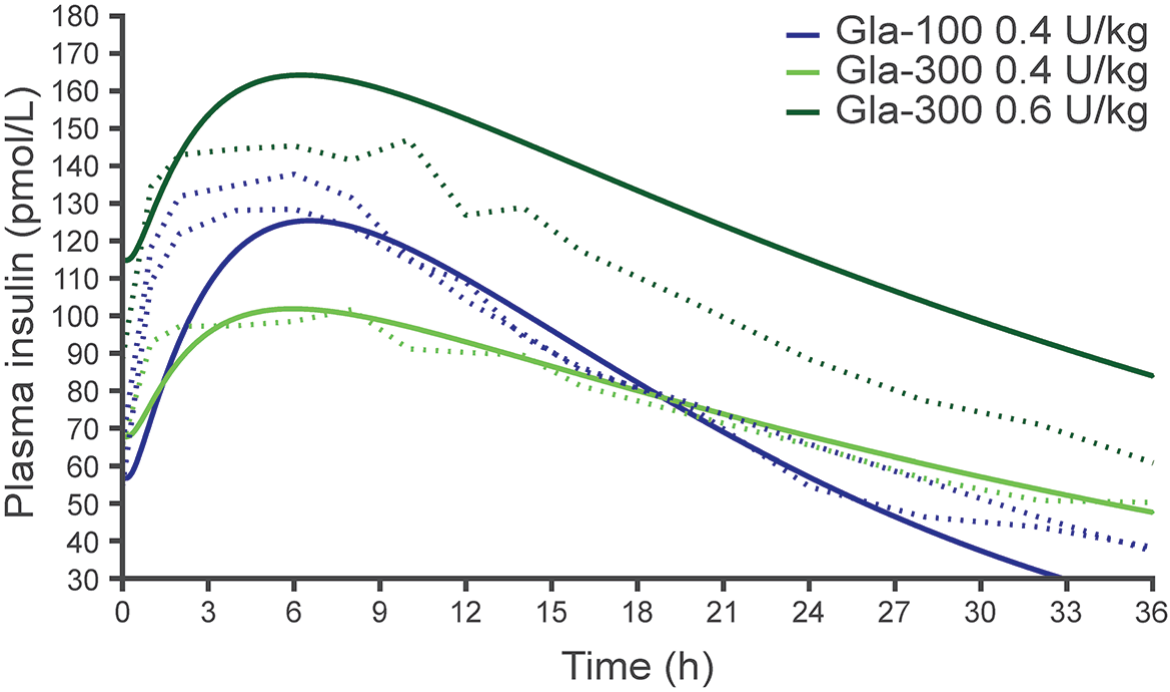
Simulation of plasma insulin concentrations over a 36-hour period, with once-daily Gla-100 or Gla-300 at steady state. Simulation based upon a person with type 1 diabetes (80 kg body weight) receiving 0.4 U/kg (2.4 nmol/kg) of Gla-100 and either 0.4 U/kg (2.4 nmol/kg) or 0.6 U/kg (3.6 nmol/kg) Gla-300 once daily. For illustrative purposes the injection at hour 24 is not modeled. Solid lines = simulated data. Dashed lines = experimental data from a euglycemic clamp study in people with type 1 diabetes [8].

By specifying an injection interval of 24 hours for 6 consecutive days, simulated single-dose PK profiles were used to generate a steady-state scenario. Simulated PK profiles during these 6 days of once-daily dosing with 0.4 U/kg (2.4 nmol/kg) Gla-300 or Gla-100 are displayed in Supplemental Figure 1 and suggest that steady state is reached after 3 days with Gla-300 and after 2 days with Gla-100. By examining the steady-state profile from day 6 independently (Figure 2), and extending it up to 36 hours post dosing, it is evident that the simulated curves reflect actual PK profiles at steady state generated during a euglycemic clamp study in people with T1DM [8]. Although the simulation appears to underestimate insulin glargine levels after Gla-100 0.4 U/kg (2.4 nmol/kg) administration at both the start and the end of the 36-hour period, and overestimates insulin glargine levels after the higher dose (0.6 U/kg (3.6 nmol/kg)) of Gla-300, the mechanistic model describes in general the PK profile. 

As evidence, MSE values for the comparison of the predicted profile with those generated from experimental data are provided in Supplemental Table 4. 

### Simulation application 1: Effect of a flexible dosing regimen 

**Figure 3.**
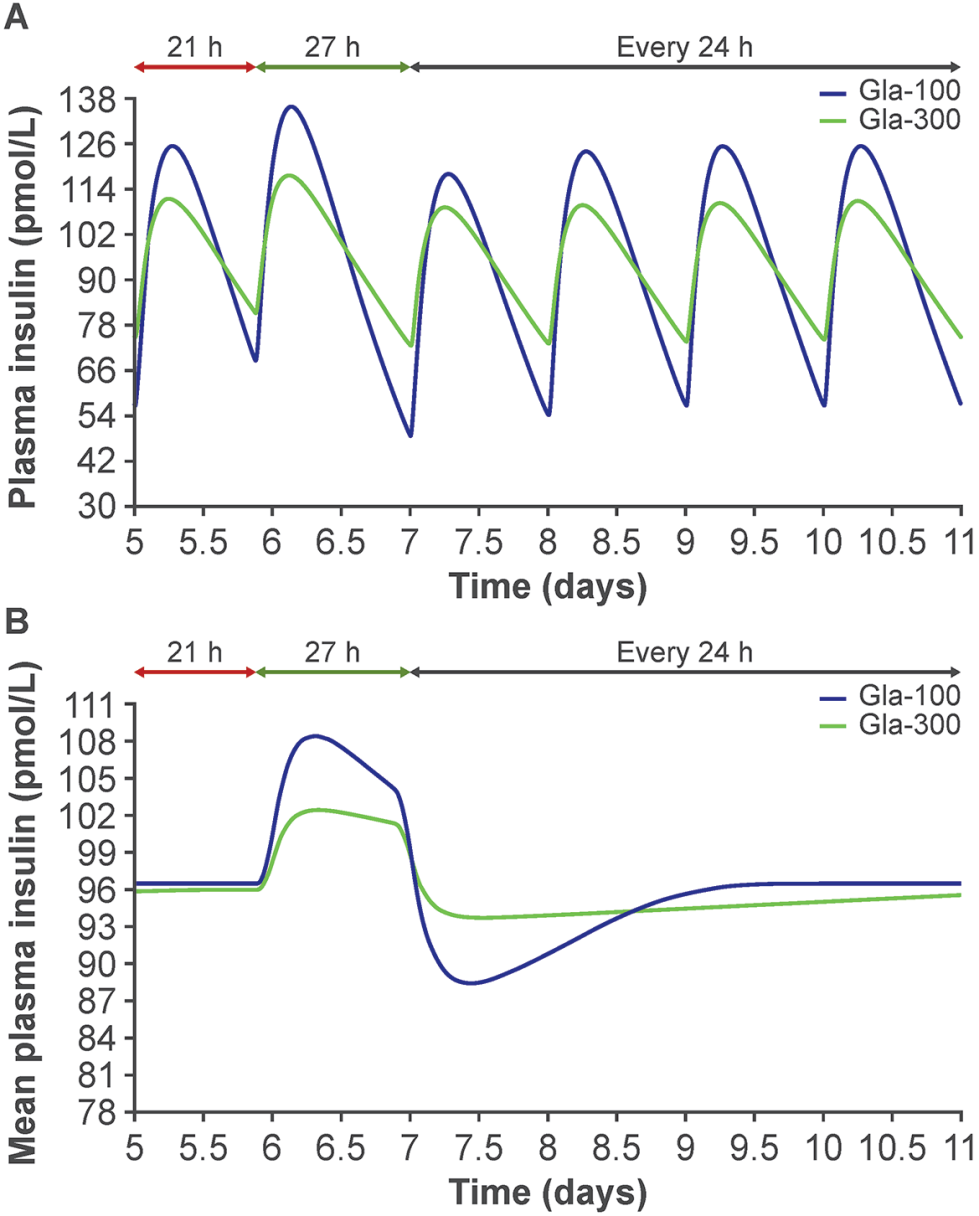
Simulated plasma insulin glargine profiles (A) and 24-hour exposure profiles (AUC*_t_*_–_24 to *_t_*) (B) with Gla-300 (0.432 U/kg (2.593 nmol/kg)) and Gla-100 (0.4 U/kg (2.4 nmol/kg)) when varying the daily injection time (scenario 1 injection intervals: 21 hours followed by 27 hours). AUC*_t_*_–24 to_ *_t_* = area under the insulin concentration-time curve in the 24 hours prior to a given time point, *t*.

When simulating a scenario in which once-daily dosing of insulin glargine is administered 3 hours earlier than normal, resulting in a 21-hour injection interval followed by a 27-hour injection interval (Supplemental Table 3, scenario 1), an effect on the PK profiles of both Gla-300 and Gla-100 can be seen (Figure 3A). However, the alteration of insulin glargine exposure is more pronounced with Gla-100 than with Gla-300 (Figure 3B). When simulating two scenarios in which the timing of injection is altered on two consecutive days, resulting in injections intervals of 27, 18, and 27 hours (Supplemental Table 3, scenario 2; Supplemental Figure 2A) or 21, 30, and 21 hours (Supplemental Table 3, scenario 3; Supplemental Figure 3A), a similar pattern is seen – plasma insulin glargine exposure with Gla-300 fluctuates less than that with Gla-100 (Supplemental Figure 2B, 3B). 

### Simulation application 2: Switching from one insulin glargine product to the other 

Using the described model, Supplemental Figure 4 shows that switching from Gla-100 to Gla-300 or vice versa, with a 7 – 8% change in daily dose, results in an initial alteration in insulin glargine exposure (a decrease in exposure when switching from Gla-100 to Gla-300, and an increase in exposure when switching from Gla-300 to Gla-100), but that steady state should be achieved ~ 3 days post switch. 

## Discussion 

### Modeling and simulation 

The mathematical models described here result in simulated PK profiles that are generally a good fit to those generated in clinical euglycemic clamp studies. However, the model does appear to slightly overestimate insulin glargine levels after administration of the higher dose (0.6 U/kg (3.6 nmol/kg)) of Gla-300. Although it was designed to capture non-specific loss of insulin glargine in the SC tissue, we hypothesize that the model described in this manuscript may have underestimated such loss for Gla-300 (and therefore overestimated Gla-300 bioavailability and plasma Gla-300 concentrations), particularly at higher doses. Future work may therefore involve identifying the source of this underestimation and adjusting the model to improve accuracy. Of note, steady-state exposure shows linear proportionality between Gla-100 0.4 U/kg and Gla-300 0.6 U/kg at an otherwise unchanged time-concentration profile [8]. The discrepancy in bioavailability estimation may also partly explain why simulating an 8% increase in Gla-300 dose resulted in the same 24-hour plasma insulin exposure as Gla-100 when treat-to-target clinical trials demonstrate that a greater increase in Gla-300 dose (a 12% increase in a patient-level meta-analysis of three studies) [16] is observed at the same level of glycemic control. The latter clinical observation may also be attributed to the different effect profile of Gla-300 vs. Gla-100, which addresses control of glucose production more effectively without promoting glucose utilization owing to less fluctuation in insulin concentration, therefore allowing higher dosing without prompting hypoglycemia [27]. Another potential limitation of the current model is the fact that the variance in insulin absorption in the studies used to develop and then validate the model was not accounted for. Including additional information such as this, as well as additional PK data from any future studies of Gla-300 and Gla-100, may help to refine the model and allow validation at different doses. 

Although there is no direct in vivo evidence that SC depots of Gla-300 and Gla-100 display the exact properties assumed in these models, the results favor the outlined model of protraction for these insulins. This modeling and simulation study suggests that the PK and PD differences between Gla-300 and Gla-100, which have been shown to translate into clinical benefits in terms of less hypoglycemia and less glycemic variability with Gla-300 vs. Gla-100 in randomized controlled trials, stem from the smaller and more compact depot of Gla-300 leading to a more gradual and prolonged redissolution of insulin glargine. The observation that the redissolution rate of insulin glargine depots is dependent upon the concentration of the injected solution is unique to this subcutaneously precipitating insulin. A number of soluble insulin products (some with alternative protraction mechanisms) have been studied as formulations with different concentrations, such as insulin lispro U200 [32], insulin aspart U20 and U200 [33], regular human insulin (RHI) U40 [34] and U500 [35], and insulin degludec U200 [36]. However, these insulins show no clinically relevant PK and hence glucodynamic differences from their U100 formulations, at least not in steady state [36]. 

One may argue that simulations demonstrating differences in the concentration profiles of insulin products that require therapeutic accumulation to achieve therapeutic effect levels can be based on terminal elimination half-life (T_1/2_). Indeed, applying the observed change from 12 hours with Gla-100 to 18 hours with Gla-300 in T_1/2_ illustrates qualitatively similar changes in insulin concentration profiles as obtained by modeling the entire precipitation and absorption process. This is different for other products that use different retarding principles and where reported T_1/2_ and time to steady-state concentration profiles are not aligned [37]. 

### Simulation applications 

Administering daily insulin injections at a fixed time and frequency can be difficult for people with diabetes [38] owing to variations in daily routine. If deviating from a 24-hour injection interval results in substantial alterations in plasma insulin concentrations, risk of hyper- and hypoglycemia is increased. Therefore, the blunting of these fluctuations with Gla-300, predicted by these simulations, has clinical implications for people with diabetes who require basal insulin treatment as they provide the option of an increased level of flexibility in injection timing to compensate for variations in daily activities. In support of the simulated data presented here, extensions to the EDITION 1 and 2 phase 3a clinical trials demonstrated that glycemic control and hypoglycemia risk with once-daily Gla-300 therapy was not compromised by participants adapting the timing of their dose by up to 3 hours either side of the 24-hour interval on at least 2 days of the week [39]. Furthermore, the potential to vary injection time with a long-acting insulin, while maintaining efficacy and safety, has also been demonstrated in studies of insulin degludec [40, 41]. 

Changing to a longer-acting insulin product for basal insulin supplementation will cause transient hypoinsulinization because the more gradual release from the SC depot necessitates extra time to achieve new steady state. Conversely, switching to a shorter-acting insulin product will cause transient hyperinsulinization and raise hypoglycemic potential as stacking occurs. Gla-100 is a widely used basal insulin analog product, many people starting treatment with Gla-300 will be switching from Gla-100. These two insulins have slightly different bioavailability and a different time to steady state, so it is suggested that when switching from Gla-100 to Gla-300, an increase in daily dose and additional time may be required to establish the targeted effect concentration, compared with altering the dose of Gla-100 [28, 29]. The model described here allowed simulation of PK profiles when individuals switch from one insulin glargine product to the other. Our simulations modeled an 8% increase in daily dose for the switch from Gla-100 to Gla-300. In contrast, switching from Gla-300 to Gla-100 would create transient elevated insulin exposure with increased hypoglycemic risk due to stacking, which is accompanied by a different time to steady state, irrespective of the difference in bioavailability. 

Although the impact of changing an insulin product on parameters such as glycemic control and hypoglycemia is best assessed in a clinical trial setting, the short deviation in plasma insulin concentrations from steady state (~ 3 days) demonstrated by these PK simulations suggest that switching from Gla-100 to Gla-300 while increasing the daily dose should be relatively trouble free. This is supported by a recent study investigating the switch from Gla-100 to Gla-300 in real-life practice [42], in which people with T1DM experienced similar glycemic control and less nocturnal hypoglycemia in the 2 weeks after switching vs. the 2 weeks before switching, despite a slight increase (~ 1 – 2 U/day) in basal insulin dose. Switching from Gla-300 to Gla-100, by contrast, has not been represented in clinical studies, but would increase hypoglycemic potential, and doses should be adapted accordingly. 

## Conclusion 

In conclusion, this modeling and simulation study provides support for the concept that the more even and prolonged PK profiles of Gla-300 compared with Gla-100 are directly attributable to a slowed surface-area-dependent release, as a result of a more compact SC depot. The modeling and simulation methods implemented here could be useful in predicting how changes in a basal insulin regimen (such as changing the interval between daily injections or switching from one insulin glargine product to the other) may affect PK profiles. 

## Acknowledgment 

Editorial support in the preparation of this publication was provided by Simon Rees, PhD, of Fishawack Communications Ltd. and was funded by Sanofi. The authors, individually and collectively, are responsible for all content and editorial decisions related to the development/presentation of this publication. 

## Funding 

This study was funded by Sanofi. 

## Conflict of interest 

Klaus Lindauer is an employee of Sanofi. Reinhard Becker was an employee of Sanofi when this work commenced, retired from the organization, and has since provided consultancy to Sanofi. 

**Table 1.** Parameter set used for modeling and simulation.

Parameter	Value	Unit	Source	Description
*k* *_FP_*	2.82	1/min	Fitted	Rate of precipitate formation
*k* *_pre_*	0.28	pmol/min×cm^2^	Fitted	Rate of glargine precipitate dissociation
*k* *_sol_*	0.34	pmol/min×cm^2^	Fitted	Rate of soluble glargine dissociation
*f* *_vol_*	3.6	NA	Set based on literature [23]	Form factor of the SC depot
*D* *_H_*	4.6860×10^−5^	cm^2^/min	Literature [4]	Diffusion constant of glargine hexamer (*H*)
*D* *_D_*	1.7926×*D* *_H_*	cm^2^/min	Literature [4]	Diffusion constant of glargine dimer (*D*)
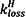	1.948×10^−5^/*V* *_Injection_*	1/min	Fitted based on bioavailability	Non-specific loss of glargine hexamer (*H*)
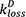	4.57× 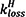	1/min	Literature [4]	Non-specific loss of glargine dimer (*D*)
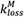	2.9×10^−3^	1/min	Literature [5]	Non-specific loss of glargine monomer (*M*)
*k* *_HD_*	0.72	1/min	Fitted	Dissociation constant: H to D
*k* *_DH_*	1.5×10^−5^	1/min	Literature [2]	Dissociation constant: D to H
*k* *_DM_*	5.81×10^−3^	1/min	Fitted	Dissociation constant: D to M
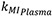	6.18×10^−2^	1/min	Literature [5]	Rate of glargine absorption into the blood
*V* *_SC_*	2.5×*V* *_Injection_*	mL	Estimated	SC distribution volume
*V* *_D_*	0.1421	L/kg	Literature [5]	Distribution volume
*k* *_Clearance_*	0.1234	1/min	Literature [43]	Constant representing the degradation of glargine
α	1.58×10^−4^	L/pmol	Literature [43]	Clearance

BM = body mass; NA = not applicable; SC = subcutaneous.

## Supplemental material

Supplemental material Supplementary Tables and Figures
