# Supplementary material for: Insulin depot absorption modeling and pharmacokinetic simulation with insulin glargine 300 U/mL
Source: Int J Clin Pharmacol Ther. 2018 Oct 26;57(1):1–10. doi: 10.5414/CP203269 (PMC6298133; doi:10.5414/CP203269)

Supplementary Table 1. Parameter estimation: parameter set obtained after fitting to clinical pharmacokinetic data, and associated mean squared error (MSE) of the predicted plasma insulin glargine concentrations.

		**Parameter set**
**Parameter**	**Unit**	
MSE	$(\frac{pmol}{L})^2$	98.6699
k_{FP}	$\frac{1}{min}$	2.8232
k_{sol}	$\frac{pmol}{min \times cm^2}$	0.341
k_{pre}	$\frac{pmol}{min \times cm^2}$	0.2846
k_{HD}	$\frac{1}{min}$	0.7156
k_{DM}	$\frac{10^{-3}}{min}$	5.8062
k_{loss}^{H}	$\frac{10^{-5}}{min \times V_{Injection}}$	1.9476

Supplementary Table 2. Calculated mean squared error (MSE) of the predicted single-dose plasma insulin glargine concentration obtained from model validation.

**Formulation**	**Dose (U/kg)**	**Study**	MSE values $(\frac{pmol}{L})^2$
Gla-100	0.4	2	31.329
Gla-100	0.4	2	43.21
Gla-300	0.4	1	1036.9
Gla-300	0.6	1	40.05
Gla-300	0.9	1	139.96

0.4 U/kg = 2.4 nmol/kg; 0.6 U/kg = 3.6 nmol/kg; 0.9 U/kg = 5.4 nmol/kg.

Supplementary Table 3. Details of three scenarios used to simulate pharmacokinetic profiles with flexible dosing intervals during a once-daily regimen.

	Dosing interval and Δ*t* (h)				
Dosing every 24 h	**24 h** Δ*t* = 0	**24 h** Δ*t* = 0	**24 h** Δ*t* = 0	**24 h** Δ*t* = 0	**24 h** Δ*t* = 0
Scenario 1	**24 h** Δ*t* = 0	**21 h** Δ*t* = −3	**27 h** Δ*t* = +3	**24 h** Δ*t* = 0	**24 h** Δ*t* = 0
Scenario 2	**24 h** Δ*t* = 0	**27 h** Δ*t* = +3	**18 h** Δ*t* = −6	**27 h** Δ*t* = +3	**24 h** Δ*t* = 0
Scenario 3	**24 h** Δ*t* = 0	**21 h** Δ*t* = −3	**30 h** Δ*t* = +6	**21 h** Δ*t* = −3	**24 h** Δ*t* = 0

Δ*t*, change from a 24-h injection interval; Green cells represent Δ*t*>0 h; Orange cells represent Δ*t*<0 h; Gray cells represent Δ*t*=0 h.

Supplementary Table 4. Calculated mean squared error (MSE) of the predicted steady-state plasma insulin glargine concentration obtained from model validation.

**Formulation**	**Dose (U/kg)**	**Time interval (h)**	MSE values $(\frac{pmol}{L})^2$
Gla-100[a]	0.4	0 to 36	182.31
Gla-100[a]	0.4	4 to 32	28.73
Gla-100[a]	0.4	0 to 24	196.99
Gla-100[b]	0.4	0 to 36	322.74
Gla-100[b]	0.4	4 to 32	76.97
Gla-100[b]	0.4	0 to 24	356.7
Gla-300[a]	0.4	0 to 36	38.3
Gla-300[a]	0.4	4 to 32	27.23
Gla-300[a]	0.4	0 to 24	43.52

0.4 U/kg = 2.4 nmol/kg.

[a]Cohort 1 and [b]Cohort 2 from a euglycemic clamp study in people with type 1 diabetes.[12]

Supplementary Figure 1. Simulated plasma insulin glargine profiles over 6 days of once-daily dosing with 0.4 U/kg Gla-300 or Gla-100.

Simulation based upon a person with type 1 diabetes (80 kg body weight) receiving 32 U (192 nmol) (corresponding to 0.4 U/kg [2.4 nmol/kg]) of Gla-300 or Gla-100 once daily. From these curves it can be estimated that steady state is reached after 3–4 days with Gla-300 and after 2 days with Gla-100.

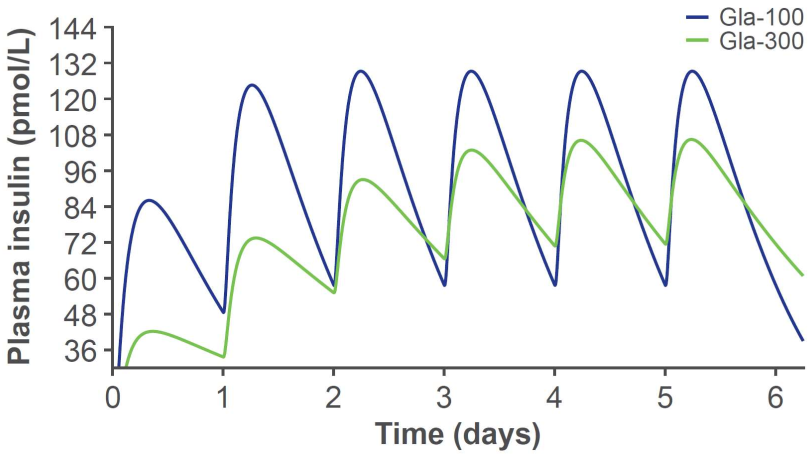

Supplementary Figure 2. Simulated plasma insulin glargine profiles (A) and 24-h exposure profiles ($AUC_{t\text{–}24\ \text{to}\ t}$) (B) with Gla-300 (0.432 U/kg [2.593 nmol/kg]) and Gla-100 (0.4 U/kg [2.4 nmol/kg]) when varying the daily injection time (scenario 2 injection intervals: 27 h followed by 18 h followed by 27 h).

$AUC_{t\text{–}24\ \text{to}\ t}$, area under the insulin concentration-time curve in the 24 h prior to a given time point, *t*.

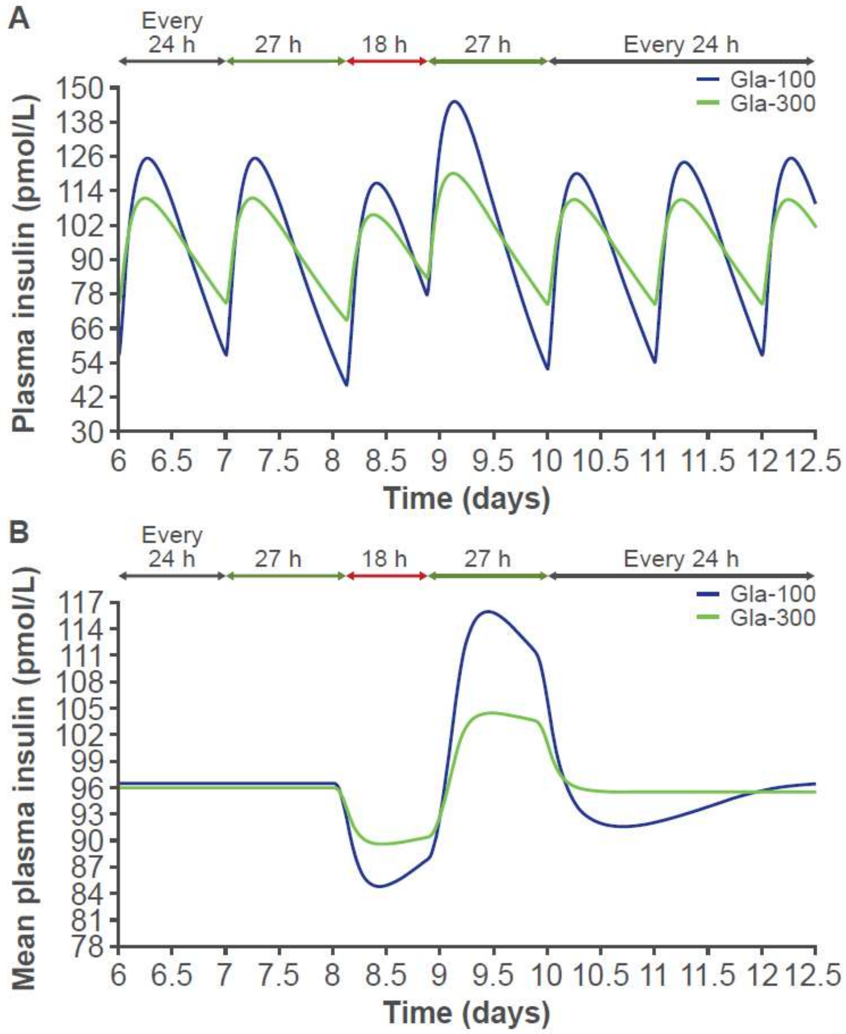

Supplementary Figure 3. Simulated plasma insulin glargine profiles (A) and 24-h exposure profiles ($AUC_{t-24\ \text{to}\ t}$) (B) with Gla-300 (0.432 U/kg [2.593 nmol/kg]) and Gla-100 (0.4 U/kg [2.4 nmol/kg]) when varying the daily injection time (scenario 3 injection intervals: 21 h followed by 30 h followed by 21 h).

$AUC_{t-24\ \text{to}\ t}$, area under the insulin concentration-time curve in the 24 h prior to a given time point, *t*.

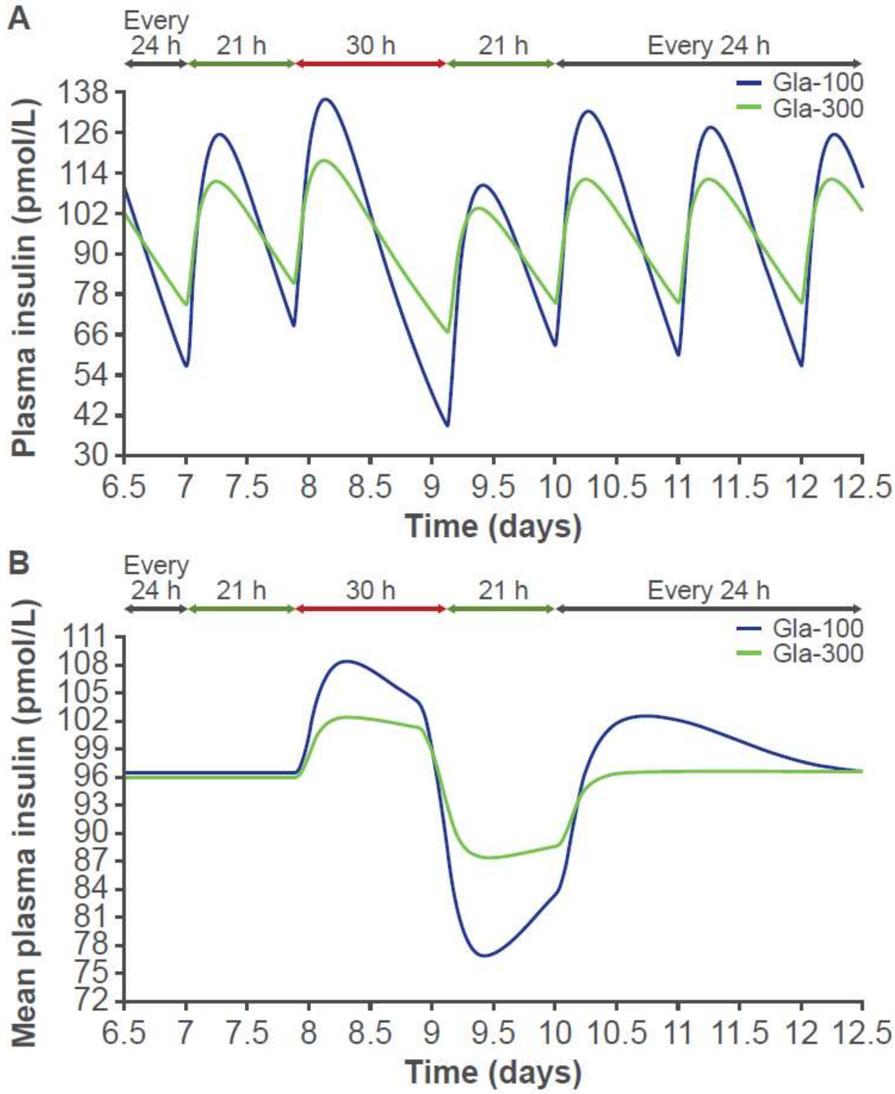

Supplementary Figure 4. Simulated plasma insulin glargine profiles (A) and 24-h exposure profiles ($AUC_{t-24\ to\ t}$) (B) with Gla-300 (0.432 U/kg [2.593 nmol/kg]) and Gla-100 (0.4 U/kg [2.4 nmol/kg]) when switching from Gla-100 to Gla-300 or *vice versa*.

$AUC_{t-24\ to\ t}$, area under the insulin concentration-time curve in the 24 h prior to a given time point, *t*.

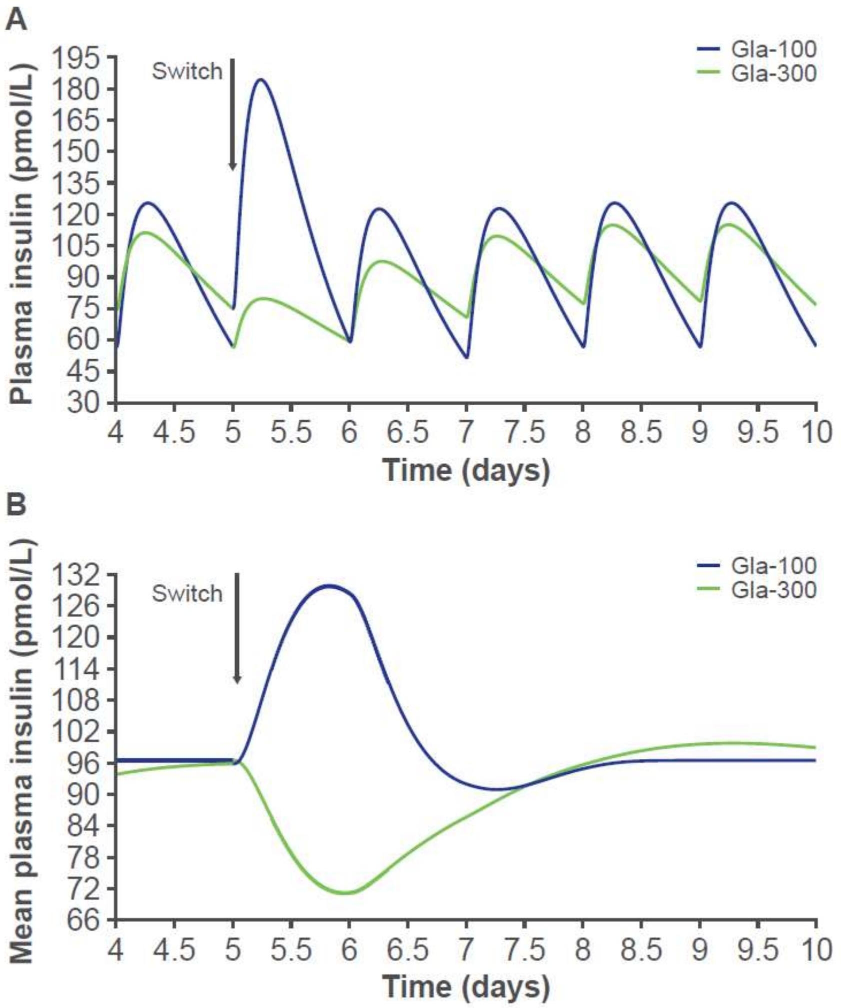

Supplement: Supplemental material [file intjclinpharmacol-57-001-S01.pdf]
